# Intracellular biocompatible hexagonal boron nitride quantum emitters as single-photon sources and barcodes†

**DOI:** 10.1039/d3nr05305a

**Published:** 2024-01-24

**Authors:** Aljaž Kavčič, Rok Podlipec, Ana Krišelj, Andreja Jelen, Daniele Vella, Matjaž Humar

**Affiliations:** a Condensed Matter Department, J. Stefan Institute Jamova 39 SI-1000 Ljubljana Slovenia matjaz.humar@ijs.si; b Faculty of Mathematics and Physics, University of Ljubljana Jadranska 19 SI-1000 Ljubljana Slovenia; c Helmholtz-Zentrum Dresden-Rossendorf e.V., Ion Beam Center Bautzner Landstrasse 400 01328 Dresden Germany; d Faculty of Mechanical Engineering, Laboratory for Laser Techniques, University of Ljubljana Aškerčeva 6 SI-1000 Ljubljana Slovenia; e CENN Nanocenter Jamova 39 SI-1000 Ljubljana Slovenia

## Abstract

Color centers in hexagonal boron nitride (hBN) have been emerging as a multifunctional platform for various optical applications including quantum information processing, quantum computing and imaging. Simultaneously, due to its biocompatibility and biodegradability hBN is a promising material for biomedical applications. In this work, we demonstrate single-photon emission from hBN color centers embedded inside live cells and their application to cellular barcoding. The generation and internalization of multiple color centers into cells was performed *via* simple and scalable procedure while keeping the cells unharmed. The emission from live cells was observed as multiple diffraction-limited spots, which exhibited excellent single-photon characteristics with high single-photon purity of 0.1 and superb emission stability without photobleaching or spectral shifts over several hours. Due to different emission wavelengths and peak widths of the color centers, they were employed as barcodes. We term them Quantum Photonic Barcodes (QPBs). Each QPB can exist in one out of 470 possible distinguishable states and a combination of a few QPBs per cell can be used to uniquely tag virtually an unlimited number of cells. The barcodes developed here offer some excellent properties, including ease of production by a single-step procedure, biocompatibility and biodegradability, emission stability, no photobleaching, small size and a huge number of unique barcodes. This work provides a basis for the use of hBN color centers for robust barcoding of cells and due to the single photon emission, presented concepts could in future be extended to quantum-limited sensing and super-resolution imaging.

## Abstract 2

Hexagonal boron nitride nanoparticles with color centers work as single-photon emitters from within live cells and enable robust photonic barcoding.

## Graphical abstract

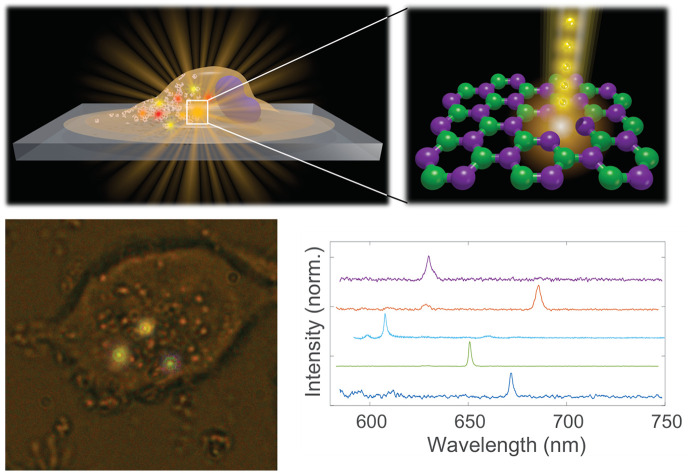

## 1. Introduction

Single photon sources,^1–6^ which are devices capable of delivering individual and temporally isolated photons with distinctive sub-Poissonian photon statistics, have been emerging rapidly in the last twenty years as suitable candidates for quantum information processing,^7^ quantum cryptography^8^ and generation of optical qubits for quantum computing,^9^ while also leading towards advancement in optical imaging and sensing, with the realization of quantum sensing,^10^ quantum and sub-shot noise imaging,^11^ super-resolution imaging^12,13^ and single molecule tracking^14^ amongst others. Artificial atomic systems in solids, such as quantum dots^15^ and color centers,^4,16–18^ are considered the leading platforms for these applications.

Amongst them, hexagonal boron nitride (hBN) is becoming one of the most widely used due to its remarkable photostability and single-photon emission at room temperature.^4,13,19,20^ hBN can host a large variety of different lattice defects, termed color centers, leading to a large range of emission wavelengths. hBN is a wide bandgap (∼6 eV) layered van der Waals crystal with alternating boron and nitrogen atoms in the hexagonal honeycomb lattice,^19,20^ with a variety of possible different structural defects (vacancies or substitute atoms),^19,21,22^ which introduce new energy levels into the system. The transitions between this newly introduced ground and excited states, referred to as zero-phonon lines (ZPLs) since they are not coupled to vibrational levels, follow the cycle of spontaneous emission in the visible spectral range with nanosecond lifetimes.^13^ As only one photon per excitation cycle can be emitted, the photons detected from such a single-photon source will be spaced in time, leading to the so-called photon antibunching.^1^

On a different note, hBN nanoparticles have also shown great biocompatibility, low cytotoxicity and high thermal and chemical stability,^23–26^ as well as the property of being biodegradable.^27,28^ Due to these reasons, hBN is widely used in different fields, from cosmetic products to medical applications.^29–31^ The main degradation product in aqueous solutions is boric acid. hBN nanoparticles are increasingly used in different biological applications, ranging from nano-carriers and drug delivery systems,^23,32,33^ biosensors,^34^ optically detected magnetic resonance (ODMR),^35^ cells and tissue imaging,^34,36^ fluorescence staining and super-resolution imaging such as stimulated emission depletion microscopy (STED) and stochastic optical reconstruction microscopy (STORM).^37–39^ However, the use of hBN color centers in biological tissues and live cells has not been studied and especially single photon emission from hBN color centers in the biological environment has not been demonstrated yet.

Important applications of different types of nanoparticles, especially fluorescent ones, are bioimaging^40^ and cell barcoding.^41^ Tracking of cells by using optical barcodes is very important for their unique identification, targeted analysis and sensing, as well as monitoring their pathogenesis.^42–45^ So far, different labels were suggested for cellular barcoding, ranging from fluorescent beads with different dyes and concentrations, to cellular microlasers and microresonators.^46–50^ Color centers, however, have not been employed yet for this application.

**Fig. 1:**
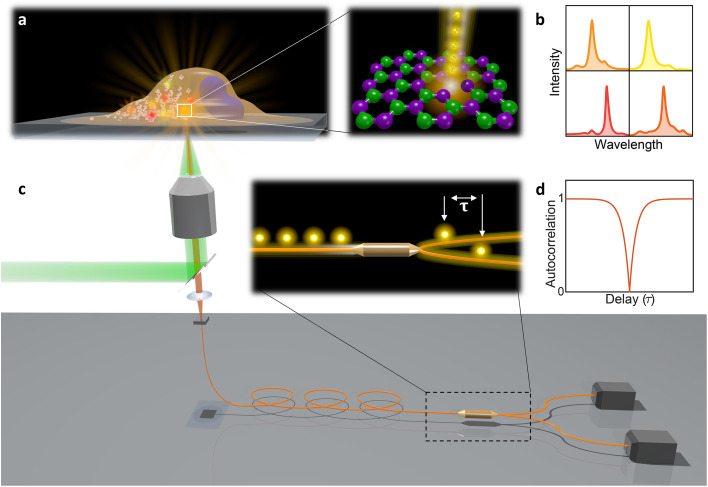
Schematic overview of the presented work. (a) A cell with integrated hBN nanoparticles. Bright localized spots indicate the emission from the color centers, which is obtained by focused laser excitation. The structure of a hBN layer consists of boron and nitrogen atoms at adjacent positions in a honeycomb lattice. Color centers, which are defects in the crystal structure, are capable of producing bright and localized single photon emission. (b) Different nanoparticles consist of different color centers leading to the emission in various wavelengths. (c) A objective focuses the laser beam on the position of the color center and also gathers the emitted light. The emitted light is then passed through a dichroic mirror and coupled into an optical fiber, which is divided into two separate arms *via* a fiber beamsplitter. The two arms are connected to time-correlated single-photon detectors, thus completing the so-called Hanbury-Brown–Twiss configuration. (d) As only one photon at a time can be generated, the beamsplitter will cause that every time only one of the detectors will detect a photon, leading to a dip in the autocorrelation function.

In this paper, hBN nanoparticles containing color centers were embedded into live cells (Fig. 1a). Stable single-photon emission from a cell is demonstrated with spectral (Fig. 1b) and temporal characterization (Fig. 1c and d) of the emission. Cellular barcoding was demonstrated as a practical application by exploiting different spectral peak central wavelengths and widths.

## 2. Results and discussion

### 2.1. Cellular uptake of hBN nanoparticles

To obtain hBN nanoparticles with color centers, a solvent-based ultrasonic exfoliation was performed^51–53^ resulting in nanoparticles dispersed in a solution. While the most known methods for introducing color centers, such as high-temperature annealing, electron beam irradiation and mechanical exfoliation,^19^ might be more suitable for other applications, the solvent-based technique was used to form a material that is suitable for the cellular uptake. Typically, solvents such as isopropyl alcohol (IPA), dimethylformamide, dimethylsulfoxide and *N*-methylpyrrolidone^53,54^ are used for exfoliation. Instead, here the exfoliation was done in a water-based solution which can be then directly added to the cell culture, making it a single-step process. Due to the hydrophobic nature of hBN^55^ a surfactant-assisted approach was used.^51,56^ A solution of poly-(vinylpyrrolidone) (PVP) polymer surfactant, which is non-toxic and is commonly applied in various biological applications,^57,58^ in phosphate-buffered saline (PBS) was used. PBS buffer enables that the final solution can be directly added to the cell media. Main results are obtained from hBN in PBS + PVP solution, but we also provide some results with hBN in IPA as it shows that different solvents can be used resulting in similar size and optical properties.

hBN nanopowder (Sigma-Aldrich, <150 nm average particle size) was added to the solvent, either isopropyl alcohol or phosphate-buffered saline (PBS) at 2.5 mg ml^−1^ ratio. In the PBS case, polyvinylpyrrolidone was added at 0.25 wt% to prevent aggregation of hBN. Exfoliation was performed with an ultrasonic bath (Elmasonic P 30 H, 120 W, 37 kHz) or a sonication tip (Cole-Parmer, 550 W, 20 kHz, with a probe model CV33, 13 mm diameter). With the bath, 2 ml dispersion was placed in a glass vial and sonicated for 6–8 h at a temperature below 60 °C. With the tip sonication, the probe was placed into the center of the cup containing 80 ml dispersion at a depth of approximately half the height of the solution. The time of sonication was 1–2 h with a 75% duty cycle. Overheating was prevented with ice around the cup. The two sonication techniques do not result in any significant difference. In the results shown the samples were produced with the ultrasonic tip, unless explicitly stated otherwise.

For the hBN nanoparticles characterization, the Thermo Fisher Verios 4G HP Schottky field emission scanning electron microscope with monochromator was used, utilizing scanning transmission electron microscopy (STEM) and scanning electron microscopy (SEM) mode simultaneously. STEM images were recorded by STEM 3+ retractable detector in bright field (BF) mode, allowing thickness contrast. SEM images were recorded by through lens detector (TLD) in high-resolution secondary electrons (SE) imaging mode, allowing us to see the topography of the sample. Combining those two detectors we get more complete morphological characterization of the sample. We were able to confirm that the process of exfoliation resulted in good dispersion, low level of aggregates forming and successful exfoliation of nanoparticles. In addition, also atomic force microscopy (AFM) analysis was performed to assess the size of the nanoparticles. The structural analysis revealed that the average size of the nanoparticles after the exfoliation was 72 ± 19 nm while their average thickness was 29 ± 12 nm (ESI Fig. 1 and 2†). Due to solvent evaporation in AFM measurements salt aggregates from the buffer up to a few micrometers in size were formed. In actual experiments however, the nanoparticles were left in the solution so salt aggregation did not occur. Negligible difference in nanoparticle size and emission properties was found between exfoliation in this solution compared to PVP solution in distilled water or isopropyl alcohol (ESI Fig. 3†). The estimated yield of nanoparticles containing color centers from this fabrication technique was approximately 0.1% corresponding to the color center surface density of approximately 0.1 μm^−2^ based on the average size of the nanoparticles. Other approaches^20,59^ have been optimized to produce higher yields. High-temperature annealing in an oxygen-rich environment^60^ showed an increase of emitters resulting in maximum density of 0.327 μm^−2^, while high-power plasma treatment followed by an annealing step and high-energy (∼MeV) electron beam irradiation yield three to eight-fold increase in emitter density.^59,61,62^ In addition, a combination of these processes with ultrasound exfoliation or optimizing the exfoliation protocol to select larger flakes,^63^ could lead to an increase in the color center density up to a maximum of ∼0.8 μm^−2^, which would lead to an average of 24 color centers per cell.

The resulting dispersion was added to a culture of HeLa cells. HeLa cells were seeded onto 35 mm glass bottom dish (Ibidi, μ-dish) and incubated overnight in the complete culturing medium (DMEM medium with 10% fetal bovine serum and 1% pen–strep) at 5% CO_2_ and 37 °C to be 20–30% confluent. The next step was adding 10–30 μl of hBN dispersed in PBS to the cell culture medium. After an additional incubation of 16–20 h the non-absorbed hBN was removed by washing with PBS three times. All the measurements with cells were performed in a microscope incubator at 37 °C and 5% CO_2_.

Study of nanoparticle distribution and internalization inside the cells was performed by a customized confocal fluorescence microscope system (Abberior) integrated with an inverted Olympus IX83 microscope. The cell membrane was labeled with fluorescent dye CellMaskOrange (Invitrogen) and the nucleus with Draq5 (Invitrogen). Hybrid simultaneous multi-channel confocal backscatter and fluorescence light detection enabled label-free imaging of nanoparticles with high contrast in 3D in addition to the precise visualization of fluorescently labelled cellular membranes and nuclei. The imaging was performed in scanning mode at 488 nm for confocal backscatter imaging of nanoparticles, 561 nm for fluorescence imaging of cell membranes and 640 nm for fluorescence imaging of cell nuclei. Multiple focal planes were imaged with Nyquist sampling applied in both lateral and axial direction for a clear visualization of nanoparticles distribution inside the cells and labelled cellular compartments in 3D. Simultaneous multichannel acquisition was achieved using notch filters in the de-scanned optical path, along with band-pass filters (470–495 nm, 580–625 nm and 650–720 nm, all from Semrock), and single-photon avalanche diode (SPCM-AQRH, Excelitas).

**Fig. 2:**
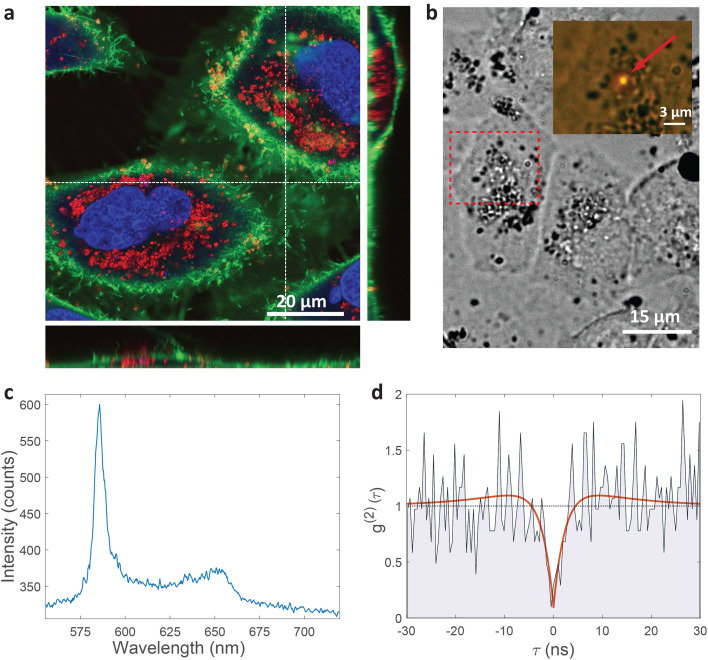
Single-photon emission from hBN nanoparticles inside live cells. (a) A confocal image of cells containing hBN nanoparticles. The signal from the cell membranes and nuclei is produced as confocal fluorescence by staining them with green and blue dye respectively, while the nanoparticles are observed as confocal backscatter signal which is visualized with red color. The majority of the nanoparticles are clearly inside the cells and are surrounding the nucleus. The main image presents a confocal imaging slice in the *xy* plane, while the bottom and side image present a cross-section in the *xz* and *yz* plane respectively, with the position denoted by dashed lines. (b) Example of a color center inside a cell excited with a green laser. Bright-field image shows the cell with internalized nanoparticles, while the inset is the fluorescence image showing the emission of the color center. (c) Spectrum of the color center shown in b, with a ZPL peak at around 590 nm. (d) Temporal autocorrelation function of the signal of the color center shows a clear antibunching deep at zero delay time, characteristic for a single-photon source. The orange line represents a fit obtained using a three-level model (eqn (2)). The ZPL from c was filtered during the detection. Autocorrelation function is not background corrected.

HeLa cells are known to be able to internalize micro and nanoparticles.^64–67^ Indeed, the majority of cells have absorbed a significant amount of nanoparticles which were visible in the cytoplasm, but not the cell nucleus (Fig. 2a). Almost no nanoparticles are present around the cells meaning that the cells are very likely to internalize the nanoparticles in their vicinity. Some nanoparticles can be seen on the membrane. The likeliest explanation is that they are in the process of internalization.

### 2.2. Characterization of single photon emission from inside cells

All measurements were performed on color centers in hBN nanoparticles internalized by live cells that were kept under physiological conditions. For optical characterization of the nature of the sources, they were analyzed both in terms of their spectral and temporal properties. The excitation of the color centers and the collection of the light was performed in an epifluorescence configuration (Fig. 1c) through a 60×, 1.0 NA water imersion objective. The excitation source was a green 532 nm CW laser with a power of 2.4 mW at the sample. The collected light was analyzed by an imaging spectrometer (Andor Shamrock SR-500i) with a 10–30 μm wide slit, a grating with 300 lines per mm and a CCD detector with a resolution of 1600 pixels. Typical exposure times were 0.5–5 s. For measuring temporal properties of the emission from the color centers, a bandpass filter (either 610 nm, 630 nm, 650 nm, 660 nm or 720 nm, all with 10 nm transmittance width) was placed in the optical path and tilted to transmit only the ZPL emission. For correlation measurements the light collected with the microscope was coupled to a multimode optical fiber (Thorlabs, 50 μm core, 0.22 NA). The Hanbury-Brown–Twiss correlation setup was realised by joining the fiber with the input arm of 1 × 2 fiber splitter (50/50 split ratio), while each output arm was coupled to one of the avalanche photodiodes (Excelitas SPCM-AQRH-13, QE > 60%, dark count rate <250 s^−1^, dead time 22 ns). The output of the avalanche photodiodes was sent to two different channels of a time-correlation unit (quTAG, 1 ps resolution). The autocorrelation function was calculated from this data with a MATLAB written code.

The color centers were easily found by a few-second laser scan of each cell. Typically, 10 times attenuated laser power (∼0.2 mW) was used for scanning the cells, resulting in intensities in the range of ∼0.1 kW cm^−2^, which is an order less than intensities that reportedly lead to photodamage in the cells.^68^ For high signal-to-noise measurements, the focus of the laser was moved both laterally and focally to achieve the highest brightness. However, most color centers were bright enough to observe them and measure their spectrum without precise laser positioning. The emission properties of the color centers show no sign of alteration due to cellular uptake. Typically a number of diffraction-limited bright spots were observed (Fig. 2b) in each cell. By analyzing their spectra it was confirmed that the majority of them possess ZPL spectral peaks (Fig. 2c). ZPLs are characteristic for single-photon color centers in hBN. However, to unambiguously prove these really are single photon emitters, the anti-bunching was quantified by the temporal autocorrelation function1
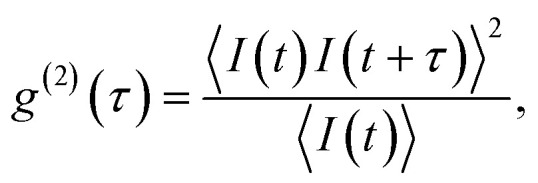
which measures the level of correlation of the signal with itself at different delays *τ* and *I*(*t*) is the signal intensity over time. The delays of single photons were measured by a Hanbury-Brown–Twiss (HBT) interferometry setup. Temporal characteristics of the color center, such as its quantum or single photon purity and lifetime, were resolved from the data by fitting a three-level model:2
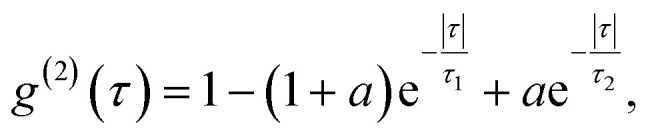
with the parameters in this equation being the bunching factor *a*, lifetime of the excited state *τ*_1_ and lifetime of the metastable state *τ*_2_.^13^ To fit the experimental results an additional parameter was introduced due to the background signal leading to a non-zero value of *g*^(2)^(0). For the example presented in Fig. 2d, the single photon purity was calculated to be *g*^(2)^(0) = 0.096, while the lifetime of excitation cycle is *τ*_1_ = 2.39 ns. A slight but distinguishable bunching with *τ*_2_ = 11.47 ns can also be observed. Presented results unambiguously prove the realization of single photon quantum emission from hBN color centers embedded inside individual cells.

A specific circumstance in our case is that the cells are a live environment, resulting in slight movement with time scales typically in the range of minutes. For this reason, additional care has to be taken for maintaining the beam position and the position of the color center aligned and maximizing the ratio of single photons with respect to background light. Our acquisition has been limited to approximately 5 min, while for longer times the laser beam would have to either be repositioned or spread into a larger spot to compensate for the cell drift. This would then further reduce the noise of the acquired signal while maintaining high single-photon purity. The cell and the cell growth medium contribute only an additional 12% to the fluorescent background compared to when not in a biological environment, so there is no significant effect on the *g*^(2)^(0) value due to cellular uptake compared to dried hBN nanoparticles on a glass surface (ESI Fig. 3†). The majority of fluorescent background originates from hBN itself. To increase the prominence of the antibunching deep, the background coming from the ambient light and unwanted fluorescence was reduced by spectral filtering of the ZPL with a filter of a 10 nm width.

To accurately characterize the ZPL peak a fitting procedure was performed to obtain the peak position and peak width. A Lorentz curve was fitted to the data in a given range around the peak which was determined based on the distance to the next local maximum. Local maxima with low prominence were neglected here. The procedure proved to be very reliable for determination of the peak position. Variation in the data interval to which the Lorentz curve was fitted, changed the ZPL position by only 0.04 nm. The determination of the width is less reliable as it depends much more on the fitting interval and the way tails of the Lorentz function are fitted. Therefore, fitting the width is affected by the presence of close-by peaks, how non-Lorentzian the shape of the peak is and the prominence as well as signal-to-noise ratio (SNR) of the peak. Based on multiple peaks and variation in fitting interval, 0.5 nm was estimated as the fitting inaccuracy of the width. In future to avoid this, the shape of the whole spectrum, also taking into account any smaller side peaks, could be used as a barcode. This would probably make the barcoding more robust and also generate more barcodes. Here, to simplify the analysis and to more easily estimate the number of unique codes, this simpler method was employed.

**Fig. 3:**
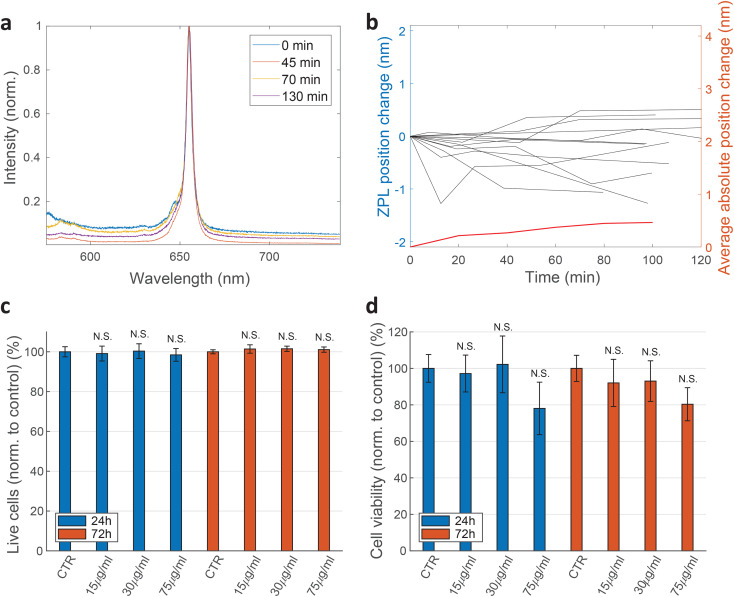
Temporal stability of emission from cells and their viability. (a) A typical example of a ZPL peak from an hBN color center inside a live cell measured for more than 2 h. (b) Temporal variation of the central position of the ZPL peak for 12 color centers in different cells. The red line denotes the average absolute shift for these 12 results (right axis). The shift is relatively small in comparison with the typical widths of the peaks. Data from samples produced with both exfoliation methods is used here. (c) Percentage of live cells and the viability of HeLa cells exposed to different concentrations of hBN nanoparticles after 24 h and 72 h, determined by flow cytometry with Annexin-V-FITC/PI staining and (d) WST-1 test. All results are shown normalised to control sample measured at the same incubation time. The results demonstrate that the cell viability is not significantly influenced by the hBN nanoparticles. N.S. indicate non-significant (*p* > 0.05) statistical difference between the viability of each test sample and the corresponding control by two-sample *t*-test. Errorbars denote the standard error obtained from 3 repetitions of the measurement with 100 000 recorded events (flow) and 6 repetitions with 100 000 cells (WST).

For practical applications, the intensity and spectral stability of the ZPL emission inside cells are very important. A typical result of a spectrum measured in time is shown in Fig. 3a and b. The changes in the positions of the ZPL peaks measured in different cells over 100 min are on average 0.46 ± 0.39 nm, which is similar to the measurement error and, additionally, of low significance considering the width of the peak on average being 4.2 nm. Also there does not seem to be a systematic drift of the peak position with some peaks shifting towards shorter and some towards longer wavelengths. Recent study^69^ has shown that the change in the refractive index from 1 to 1.52 as the silica coating is deposited on the nanoparticles, induces a ZPL shift of approximately 2.8 nm. In our case, however, the effect of the changing cellular environment should be much smaller, as firstly, there is only the change in the surrounding medium and no chemical bounding is present, secondly, the defects are not necessarily on the surface and, thirdly, the change in refractive index is much smaller (typically in the range of 1.33–1.37 ^65^). Extrapolating the findings from ref. 69 to this refractive index change would mean a ZPL shift of ≈0.22 nm which is both consistent with our results and also within the measurement error leading to the conclusion that the emission of color centers is mainly unaffected by refractive index change due to the variations in cellular environment. For peaks analyzed in Fig. 3b also their FWHM value change over time was characterized (ESI Fig. 4†). On average the peak FWHM changes for 0.56 ± 0.56 nm which is more or less comparable to the measurement and fitting error. Larger discrepancies are present in some examples but those come mainly from the peak intensity change and therefore change in SNR which influences how reliably the width can be fitted. For future practical uses, the fitting procedure for both peak position and width could be further improved. Both the peak wavelength and the FWHM value, therefore, did not show any significant temporal variations.

The emission intensity of a single color center inside a cell decreased significantly or even became lost over a few minutes since the nanoparticles were moving relative to the excitation laser beam. However, if the laser was repositioned on the color center, the emission intensity was the same within 10% over several hours. The same hBN nanoparticles deposited directly to a glass substrate showed the same level of stability with continuous illumination for at least a few hours (ESI Fig. 5†). Sometimes discrete steps in the intensity and wavelength were observed over time, although blinking, where the intensity would drop to zero, was rarely observed.

hBN nanostructures are known to be biodegradable in aqueous media. This feature, along with their biocompatibility and non-toxic nature, makes them suitable for a variety of medical applications. The biodegradation could however lead to loss of ZPL emission from hBN nanoparticles inside cells. Reportedly, only a few percent of hBN nanomaterial is lost after one month in water-based solutions.^27,28^ This time is much longer than the time of typical experiments on cells. Throughout this study, no reduction in the number of ZPLs inside cells over several days was found.

The effect of the exfoliated hBN nanoparticles on the cells was examined with a set of different measurements. Flow cytometry analysis of cell apoptosis and necrosis by double staining with Annexin-V-FITC and propidium iodide (PI) was performed. HeLa cells were seeded in a 24-well plate at the density of 50 000 cells per well for 24 h before hBN nanoparticles were added in a concentration of 15, 30 or 75 μg mL^−1^. After an additional 24 h and 72 h incubation time with the nanoparticles, the samples were prepared for apoptosis/necrosis analysis according to manufacturer instructions (FITC Annexin V Apoptosis Detection Kit I, BD Pharmingen™). As a positive control for apoptotic cell death, 1 μM Staurosporine (STS) was used which successfully induced apoptosis after 4 h of incubation with a statistically significant difference compared to the control sample. Cells were analyzed by a flow cytometer Attune™ NxT using FlowJo software. Three biological repetitions were performed for each condition and 100 000 events were recorded per tube. Only single cells were gated for fluorescence analysis. Cells that were both PI negative and Annexin-V negative are shown as viable (Fig. 3c). Cells that are only Annexin V positive are considered as early apoptotic, cells that were positive to both PI and Annexin V are considered late apoptotic and cells that are only PI positive are considered necrotic. The extent of each cell death at different nanoparticle concentrations is represented in ESI Fig. 6.† Two-sample *t*-test was performed for statistical analysis of the results. The results show negligible statistical difference in the percentage of viable cells between control samples and samples treated with nanoparticles.

Cell viability was also determined by WST-1 test. HeLa cells were seeded at the concentration of 100 000 cells per well in a 96-well plate (TPP) 24 h before they were exposed to different concentrations of hBN nanoparticles (0, 15, 30, 75 μg mL^−1^). After 24 h and 72 h incubation with nanoparticles, WST-1 reagent was added according to a manufacturer instructions (WST-1 Cell Proliferation Reagent, Abcam). Samples were analysed on a microplate reader Tecan Infinite M1000 Pro. The absorbance of each sample was measured at 450 nm. First controls were performed by incubating only the cell medium with the corresponding concentrations of nanoparticles. The values obtained from these controls were then subtracted from their corresponding cell samples. Final results are normalised to the second control sample (cells without nanoparticles). Again, no statistically significant discrepancies are observed (Fig. 3d).

The effect of the hBN on cell viability was also measured by counting the number of live and dead cells by a live-dead cell viability assay, performed by staining the cells with Hoechst 33342 (Invitrogen) and Propidium iodide (Sigma Aldrich). The majority of cells (96.5 ± 1.3%) were viable 1 day after adding the hBN dispersion (final concentration 75 μg mL^−1^) to the cell culture (ESI Fig. 7†). The viability is statistically not significantly different compared to cells without hBN (96.5 ± 1.1%).

Lastly, the effect of the hBN nanoparticles on the cell proliferation rate was studied. The proliferation over 24 h was measured for different concentrations of hBN nanoparticles and compared to the values obtained for the non-treated cell sample (ESI Fig. 8†). The values were the same within the statistical error and show that internalization of hBN nanoparticles results in a negligible effect on cell proliferation rate.

To sum up, none of the biological assays shows any statistically significant difference in cell viability and proliferation rate at 24 h or 72 h incubation time with any of the nanoparticle concentrations compared to the controls. There is only a slight decrease in cell viability at the highest nanoparticle concentration (75 μg mL^−1^) used in our study, although still statistically insignificant. Therefore, it can be concluded that the exfoliated hBN nanoparticles are indeed biocompatible and non-toxic, which is in agreement with other studies.^34^ While in those studies mainly untreated hBN was used, we have shown that also solvent exfoliated hBN does not reduce the cell viability. The concentration of 75 μg mL^−1^ hBN used in the current study could be significantly decreased to have no impact on cell viability. It could be possible to decrease the concentration of the nanoparticles by a factor of 8, by using known methods that produce more color centers per nanoparticle, as described in a previous section. The required concentration of nanoparticles would be only 9 μg mL^−1^ to yield 3 color centers per cell. Lastly, many hBN nanoparticles could be encapsulated in a single larger nanosphere of an inert material such as polystyrene or a biodegradable polymer by using standard encapsulation methods,^70^ completely eliminating the physical contact between the hBN nanoparticles and the cell. In this case, only one such inert nanosphere per cell could be enough for barcoding.

### 2.3. Cellular barcoding using single photon sources

Cellular barcoding is demonstrated as a very promising application of intracellular hBN color centers. To tag as many cells as possible with unique optical barcodes, light sources with many different distinguishable spectra are required.^41^ hBN color centers are perfectly suited for this purpose because they have a broad range of possible emissions and small size, which enables the integration of multiple color centers into a single cell with minimal invasiveness. Due to their quantum nature we name them Quantum Photonic Barcodes (QPBs).

**Fig. 4:**
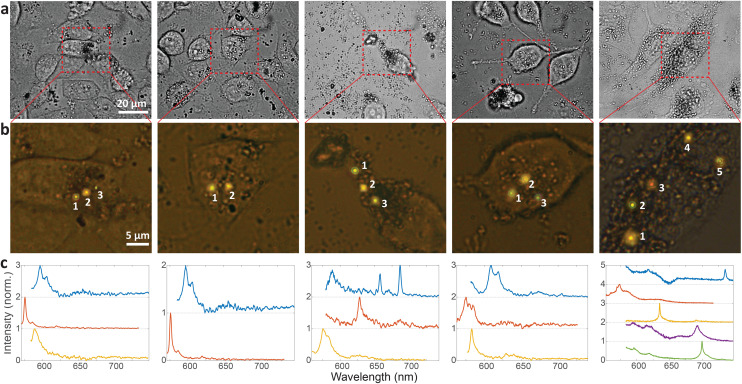
Cell barcoding with distinguishable spectral properties of hBN color centers. (a) Bright field images of 5 different cells containing hBN nanoparticles. (b) Fluorescent images of zoomed in regions denoted with the dashed rectangular shape in a. The emission from the color centers is overlaid to the bright field images of the cells. (c) Normalised and background-corrected spectra from the corresponding color centers.

**Fig. 5:**
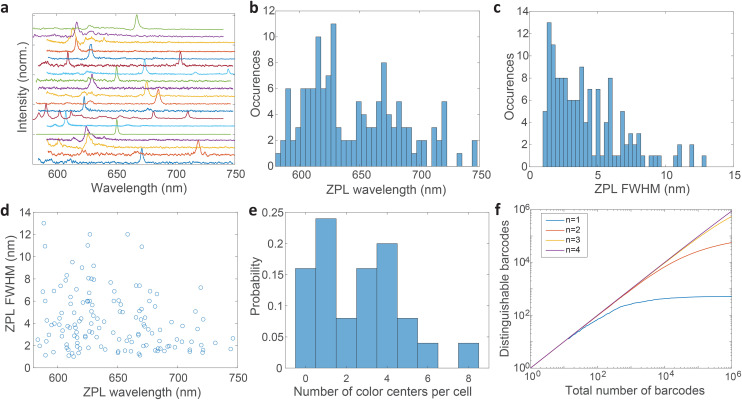
Statistics of ZPL spectra from hBN color centers. Data from both exfoliation methods and both solutions is gathered. (a) Representative spectra of 19 different color centers taken on nanoparticles deposited on a glass surface. The spectra are background corrected, normalised and increasingly translated for clarity. (b) The distribution of central wavelengths of ZPL peaks. (c) The distribution of the ZPL peak widths (FWHM). (d) The correlation between the ZPL peak wavelengths and their widths. The sample size is 139 for b, c and d. (e) The distribution of the number of color centers inside each cell (*N* = 25). (f) Monte Carlo calculation of the number of distinguishable barcodes as the total number of randomly picked ZPLs is increased. Both spectral peak wavelengths and widths are taken as independent variables to generate a barcode. The calculation is performed for cells containing different numbers of color centers (*n*).

Emission from 5 representative cells containing hBN nanoparticles is shown in Fig. 4. The emission was measured from all color centers within each cell (Fig. 4a and b). From the color of the color centers and the spectra, it can be already seen that the color centers emit at different wavelengths. It has to be noted, however, that the emission color of the color center seen in Fig. 4b does not necessarily coincide with its ZPL peak (Fig. 4c) as there is an additional fluorescent background present. In order to provide a more quantitative estimate regarding the number of possible distinguishable spectral combinations and therefore unique QPBs, the statistics of ZPL emission from a large sample (*N* = 139) of color centers were gathered (Fig. 5). Results contain data from nanoparticles dissolved in both solvents and exfoliated with both ultrasonic techniques. No difference between these cases was observed.

Most spectra from single color centers show one prominent peak corresponding to ZPL (Fig. 5a). Sometimes multiple peaks were observed in a single spectrum, due to the close proximity of multiple color centers that cannot be resolved spatially. In all likelihood, the color centers originate from different nanoparticles rather than from a single nanoparticle possessing multiple defects. Due to the fact that only a small fraction of nanoparticles incorporates a defect it seems highly unlikely that nanoparticles with multiple defects would regularly be present but we cannot really provide a proof for that nor is it really important in the scope of this work. If more spectral peaks were present in a single spectrum, only the strongest was taken into account for further analysis as it cannot be reliably determined whether the weaker peaks are actual ZPLs and their width is much harder to accurately define. However, by taking into account all of the peaks, the number of unique barcodes would actually be further increased but for the cost of making the analysis far less transparent and understandable. The results can therefore be interpreted as the conservative minimum number of barcodes, while in reality it can be greater.

The distribution of central wavelengths of ZPL peak positions shows a wide range in the interval 580–750 nm (Fig. 5b). Almost all wavelengths in this range are present, which is beneficial for barcoding to have as many distinguishable peaks as possible. There are, however, two main groups of wavelengths, centered at ≈620 nm and ≈680 nm, which are more frequently observed. This is in agreement with two groups into which the emissions can be classified according to the literature.^4,21,51^ The distribution of full width at half maximum (FWHM) of ZPLs shows that most peaks are narrow (1–3 nm), while the number of very wide peaks is smaller (Fig. 5c). The FWHM distribution has a median of 3.59 nm. There is almost no correlation between the ZPL peak wavelengths and their widths (Fig. 5d), with only a small correlation coefficient of −0.233. This means that the central wavelength and the width can be taken as two independent parameters for the purpose of creating a large number of barcodes.

The number of color centers in each cell was counted and the distribution was plotted (Fig. 5e). Only 15% of cells did not contain any color centers, while others contained up to 8 color centers. On average cells contained 3 color centers (median value). These color center numbers were achieved by a relatively small amount of hBN material added to the cell culture medium (0.005 wt%) which is only 3–10% of the cell mass in the culture. However, not all these particles are uptaken. The number of color centers could be therefore much increased if necessary, by increasing the amount of material added to the cells and the incubation time.

Based on the wavelength range of the ZPL emission peaks, their widths and the number of color centers in each cell the maximum number of unique barcodes that can be generated was calculated by a simple combinatorial calculation. For ZPL wavelength a uniform distribution spanning from the minimum ZPL wavelength (580 nm) to the maximum wavelength (750 nm) was assumed. Two peak wavelengths were considered distinguishable if separated by more than their average FWHM value. Given the median of FWHM values being 3.6 nm, in the wavelength range of 170 nm approximately 47 peaks can be distinguished.

Similarly, for different FWHMs, a uniform distribution was assumed in the range from 1 nm to 6 nm. Wider peaks were not taken into account, since they are relatively rare. Two peak widths were considered distinguishable if different by more than the width fitting error and the temporal variation of the width which is approximately 0.5 nm (Fig. 3b). Therefore, the number of distinguishable widths is approximately 10. Since the ZPL wavelength and ZPL width are uncorrelated, the total number of possible unique states that can be distinguished from a single ZPL is 470. This is significantly more than is achievable by fluorescent dyes and is comparable to state-of-the-art barcoding using microcavities and microlasers,^41,46^ with the advantages of the hBN nanoparticles being significantly smaller and easier to produce as well as completely nontoxic and biodegradable. Further, by taking into account that the cells in our case contain on average 3 color centers, then the number of possible unique barcodes is 
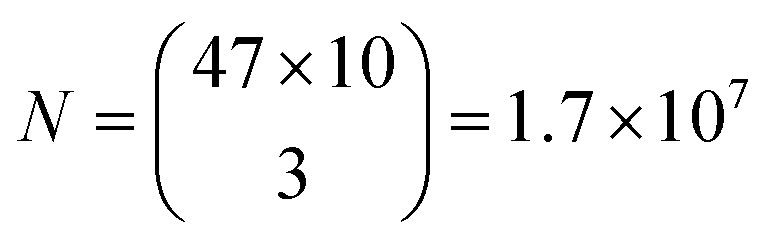
.

This result is the maximum possible distinguishable barcodes, but in practice, by labeling cells, ZPLs are randomly picked. It can happen that two ZPLs will not be distinguishable, by either the two peaks being too close to each other or the FWHM being almost the same. Also if the spectral peaks are not perfectly spaced by one FWHM, then less of them can be fitted within the wavelength region. Therefore, more useful information is obtained by counting how many randomly generated barcodes are on average distinguishable. Based on this result it can be learned how many cells, labeled with this technique, will be distinguishable in practice from the entire sample. This number is estimated by a Monte Carlo model where the barcodes are randomly drawn. The peak positions and the widths are simulated based on actually measured distributions (Fig. 5b and c). Each randomly generated barcode is compared with previously generated ones to determine whether they are distinguishable. The number of distinguishable barcodes as a function of the total number of randomly picked ones is plotted (Fig. 5f). When the number of randomly picked barcodes is small, almost all of them are distinguishable since there is a small probability of two being the same. But as the number of randomly picked barcodes increases the number of distinguishable barcodes converges towards a plateau. For instance, by having a sample of 10^5^ cells with 3 color centers per cell, 85% of them will be distinguishable (ESI Fig. 9a†). By increasing the number of color centers per cell, the number of distinguishable barcodes increases nearly exponentially (ESI Fig. 9b†). With 4 color centers per cell over 10^6^ cells would result in the same 85% distinguishability level, or if a smaller number of cells is used, for example 10^5^, a distinguishability level of 96% can be achieved (ESI Fig. 9c†). Therefore by modestly increasing the amount of hBN material added to the cells, the barcoding capability is nearly unlimited and certainly enough for more or less any advanced practical cell application, such as flow cytometry,^71^ which would greatly benefit from cell barcoding.^72^

## 3. Conclusions

In conclusion, we have demonstrated intracellular single-photon emission by designing a simple procedure of cellular internalization of hBN nanoparticles with bright and stable color centers. In future, this concept can directly lead to the implementation of a variety of quantum imaging and sensing techniques at a cellular level, similarly as in the case of NV nanodiamonds which are increasingly used for variety of bioimaging applications.^73^ The antibunching property of single-photon emission has already been exploited to increase the sensitivity of optical measurements as well as spatial resolution. The reduction of shot noise by imaging with these sources leads to sub-shot noise imaging^74–76^ and determination of weak signals with higher precision, for instance, measurements of weak absorption.^1^ Further, super-resolution imaging can be performed by temporal analysis of the emitted signal. Measuring single-photon correlations can reveal the presence of more sources whose centers are separated less than the diffraction limit.^74,76^

Moreover, due to high photostability and a broad spectral range of hBN color centers, they may be used to complement or replace standard fluorescent dyes used for instance in STED, STORM and single-molecule localization microscopy (SMLM).^12,38,39^ In addition, spin-active defects in hBN were reported, serving as a platform for quantum, spin-based, sensing, for instance for ODMR.^35^ Combining quantum single-photon emission and superresolution imaging with sources embedded in the cellular interior is therefore a design that may in future lead to superior bioimaging. This work provides a general demonstration of the possibility of single-photon emission in biological environments, namely inside cells, providing a simple, robust and repeatable process of cellular internalization. Such a concept, may inspire additional research about exploiting the practical advantages of single-photon emission in biological research.

Last but not least, by using QPBs we have also proven robust long-term barcoding of individual cells. The number of unique barcodes is significantly higher than distinguishable colors achievable by using standard fluorescent probes and is comparable to other state-of-the-art barcoding methods, such as graphical microbarcodes^77^ and microcavity- or microlaser-based barcodes,^47,48,78,79^ but with a number of significant advantages. Firstly, in hBN color centers the barcode is encoded in the spectrum, which enables easy readout independent of the orientation of the emitter and is relatively insensitive to autofluorescence and scattering. Secondly, the emission is insensitive to external factors, such as temperature, pH, refractive index and mechanical forces. Thirdly, the majority of other optical barcoding devices are based on fluorescence and contain organic dyes, which are prone to photobleaching, while hBN color centers exhibit no photobleaching and therefore higher temporal stability. Fourthly, QPBs are significantly smaller than most others, with the size of single nanoparticles is 70 nm with a thickness of 30 nm. This results in the possibility of integrating more particles within a cell, therefore increasing the number of distinguishable cells. Additional advantages include easy, cheap and scalable production by a single-step process. Further, great biocompatibility and long-term biodegradation enable safe use in biological organisms.

In conclusion, the color center based barcodes demonstrated here are the ultimate candidate for optical barcoding in biological and cellular environments. Combined with the single-photon emission of the color centers, the presented capabilities could be further extended into a powerful multimodal method for labeling, identifying and tracking a large number of individual live cells with simultaneous high resolution, low noise imaging.

## Data availability

The data that support the findings of this study are available from the corresponding author upon reasonable request.

## Conflicts of interest

The authors declare no competing financial interest.

## Supplementary Material

NR-016-D3NR05305A-s001
