# Supplementary material for: Intracellular biocompatible hexagonal boron nitride quantum emitters as single-photon sources and barcodes
Source: Nanoscale. 2024 Jan 24;16(9):4691–702. doi: 10.1039/d3nr05305a (PMC10903403; doi:10.1039/d3nr05305a)
Supplement: NR-016-D3NR05305A-s001 [file NR-016-D3NR05305A-s001.pdf]

Electronic Supplementary Material (ESI) for Nanoscale.
This journal is © The Royal Society of Chemistry 2024

Supplementary Information

Intracellular biocompatible hexagonal boron nitride quantum emitters as single-photon sources and barcodes

Aljaž Kavčič[1,2], Rok Podlipec[1,3], Ana Krišelj[1], Andreja Jelen[1], Daniele Vella[4], and Matjaž Humar*[1,2,5]

[1] *Condensed Matter Department, J. Stefan Institute, Jamova 39, SI-1000 Ljubljana, Slovenia*
[2] *Faculty of Mathematics and Physics, University of Ljubljana, Jadranska 19, SI-1000, Ljubljana, Slovenia*
[3] *Helmholtz-Zentrum Dresden-Rossendorf e.V., Ion Beam Center, Bautzner Landstrasse 400, 01328 Dresden, Germany*
[4] *Faculty of Mechanical Engineering, Laboratory for Laser Techniques, University of Ljubljana, Aškerčeva 6, SI-1000 Ljubljana, Slovenia*
[5] *CENN Nanocenter, Jamova 39, SI-1000 Ljubljana, Slovenia*
**E-mail: matjaz.humar@ijs.si*
Keywords: hexagonal boron nitride, single-photon source, color centers, live cells, spectral barcodes, cell tagging

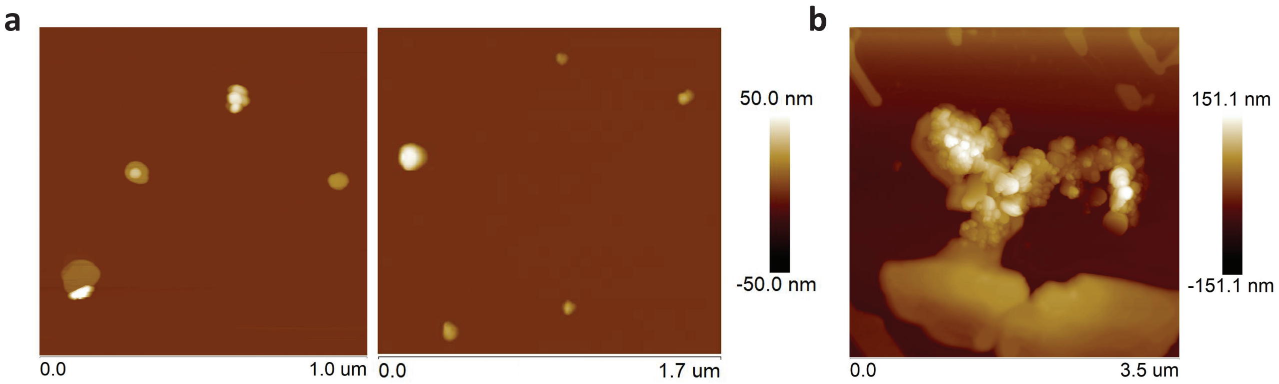

Figure 1: AFM size and structure analysis of solvent exfoliated hBN nanoparticles. **a)** AFM image of hBN nanoparticles exfoliated in IPA. Isolated particles can be obtained and their dimensions can be easily reproduced. The lateral size of the nanoparticles is $72 \pm 19\,\mathrm{nm}$ while their thickness is $29 \pm 12\,\mathrm{nm}$ ($N = 80$). **b)** AFM image of hBN nanoparticles exfoliated in PBS+PVP solution as they were used for cellular internalization. Due to evaporation larger aggregates of salts from the buffer are formed making it problematic to observe individual nanoparticles. Nevertheless, in the thickness map sharp steps can be observed very clearly, defining the edge of given nanoparticles. So, from measuring step to step distance and step height we can resolve characteristic size and thickness of the nanoparticles quite reliably. The lateral size of nanoparticles is $70 \pm 12\,\mathrm{nm}$ while their thickness is $29 \pm 7\,\mathrm{nm}$ ($N = 29$). The size of the nanoparticles is therefore compatible between exfoliation in different solvents. The only difference is the size of the aggregates which form if the solvent is left to evaporate. In the typical measurements, however, the solvent did not evaporate as the solution is directly added to the cells, so the level of agregates forming is even lower.

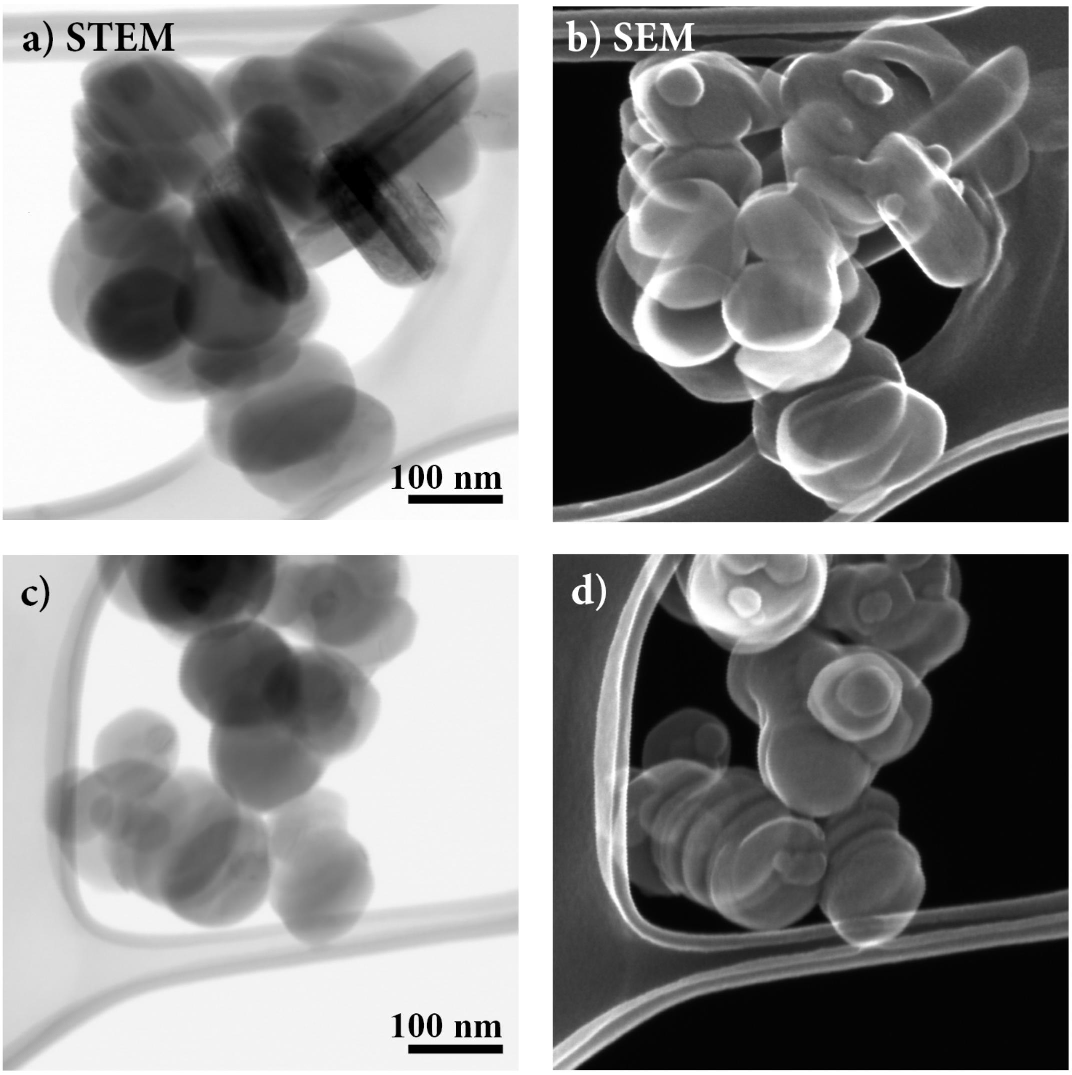

Figure 2: STEM and corresponding SEM images of hBN nanoparticles exfoliated in IPA. Two different regions are shown with the left images (**a** and **c**) acquired in STEM mode and right images (**b** and **d**) in SEM mode simultaneously, in order to distinguish individual nanoparticles. STEM images of hBN nanoparticles at different samples of the same container confirm that the nanoparticles shape is trully platelike (2D) and the sizes agree with previous analysis performed with AFM. The samples were prepared with the same exfoliation procedure and identic concentration as for the AFM experiments. The drop of the solution was placed on the conventional Cu lacey carbon TEM grid and left for the solvent to evaporate to be vacuum compatible. Thermo Fisher Verios 4G HP Schottky field emission scanning electron microscope with monochromator was used. The **a** and **c** images were recorded using STEM detector in BF imaging mode. Corresponding **b** and **d** images were recorded using TLD detector in SE (Secondary Electrons) imaging mode.

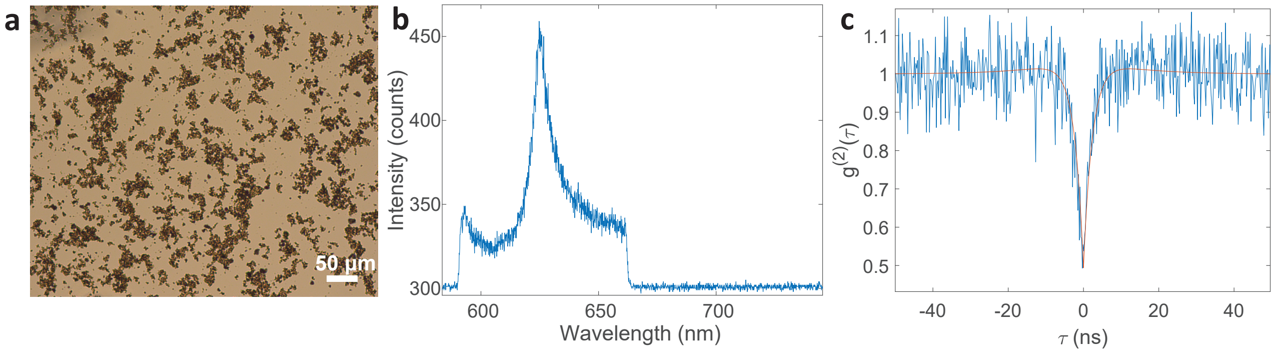

Figure 3: Color centers in hBN nanoparticles exfoliated in IPA via ultrasonic bath and drop-casted on a glass slide. **a)** A bright field image of the exfoliated nanoparticles on a glass substrate. **b)** ZPL spectrum of a characteristic color center. **c)** The corresponding $g^{(2)}$ function proving the single photon nature of the emission. The measurement is slightly less noisy than in the case of cells, as the sample is fixed and longer acquisitions can be taken.

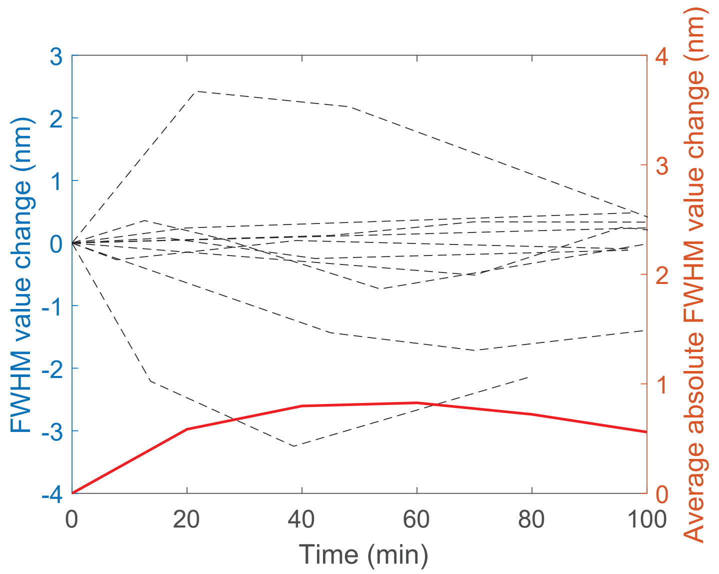

Figure 4: Temporal variation of the width of the ZPL peak for 10 color centers in different cells. Data from samples produced with both exfoliation methods is used here. The red line denotes the average absolute shift for these 10 results (right axis). On average the peak FWHM changes for 0.56 ± 0.56 nm which is more or less comparable to the measurement and fitting error and no tendency for either peak broadening or narrowing is present. The bigger discrepancies are mainly the effect of peak intensity change, therefore a reliable fit of the width, strongly dependent on the tails of the Lorentz function, cannot be performed.

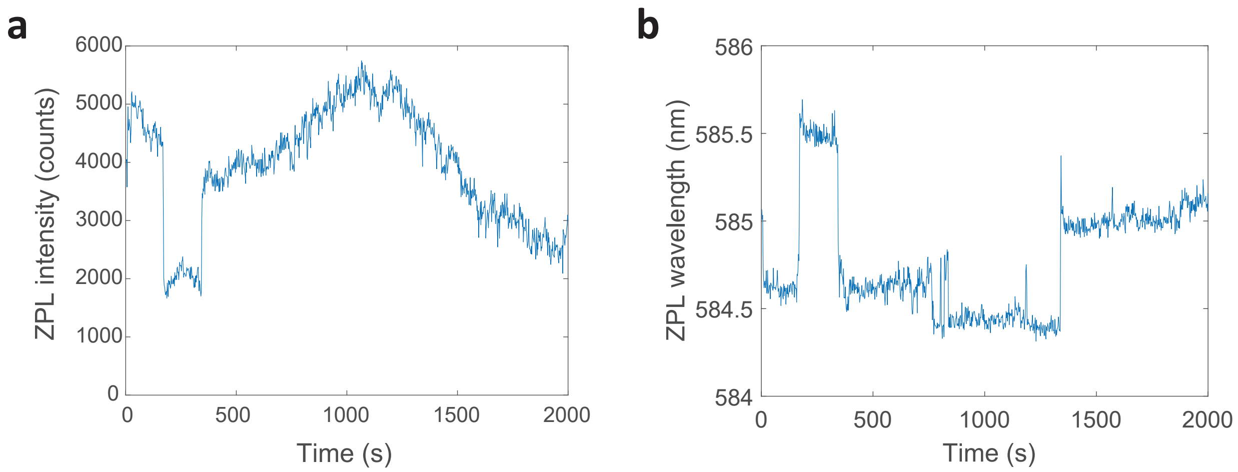

Figure 5: Temporal behavior of the color centers in general shows fairly steady behavior while some interesting discrete jumps, both in intensity and emission wavelength, occur from time to time. Although in the scope of this work this characteristic is not really important its discrete nature again points towards the quantum nature of the sources. We, however, have not been devoted to a thorough analysis on the presented behavior. To our understanding What causes this shift and whether it could in fact be bigger is not even fully understood yet as not all the possible defects that can exist in hBN and their properties have been properly investigated. **a)** Intensity changes over long time can be considered reasonably stable. Blinking was observed only in $\approx 5\%$ of the investigated color centers. **b)** The emission wavelength is also very stable. Some discrete changes, which are usually accompanied by simultaneous intensity changes, are small. No difference regarding this type of behavior for hBN nanoparticles drop-casted on a glass slide and those internalized by cells was observed. For barcoding, the intensity variations do not present a problem since the ZPLs are very prominent and the intensity remains high enough for detection, while wavelength variations are small ($< 1\,\mathrm{nm}$) in comparison with typical ZPL width (median 3.59 nm).

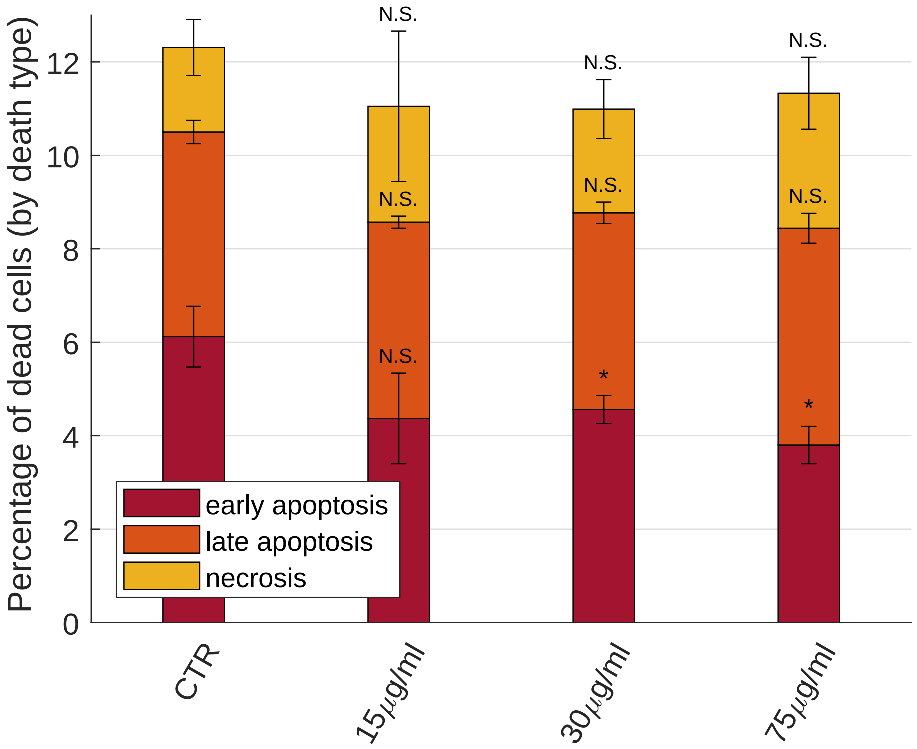

Figure 6: The occurrence of different types of cell death of HeLa cells exposed to different concentrations of hBN nanoparticles measured after 72 h of incubation. The data was collected from flow cytometry with Annexin-V-FITC/PI staining. The results were analyzed via a two-sample t-test where N.S. indicates no statistically significant difference ($p > 0.05$) and * indicates a statistically significant difference with $p < 0.05$. Each result is compared to its corresponding control.

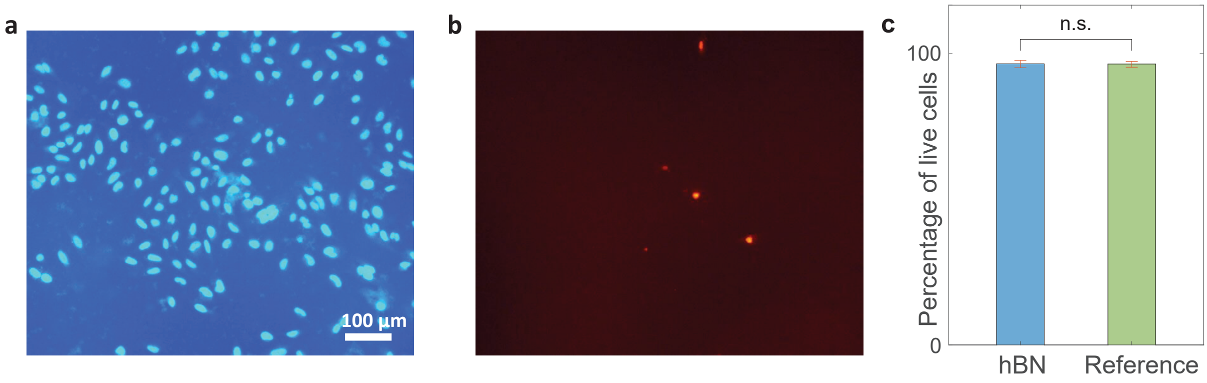

Figure 7: The effect of the hBN on cell viability was measured by counting the number of live and dead cells by Hoechst33342-PI double staining method. **a)** Typical image of a sample with the nuclei stained with blue dye (Hoechst33342). In this way, all of the cells in the sample can be identified. **b)** Red dye (propidium iodide) that specifically stains only dead nuclei was used to identify only dead cells. In this way, the percentage of live cells can be determined. Figures presented here are just the characteristic examples, for the actual analysis a total of $N = 1070$ cells was used. **c)** Percentage of live and dead cells after 24 h in a sample with and without the addition of hBN dispersion. Based on the Two-sample t-test it can be concluded that the two distributions have no statistically significant means (n.s., $p > 0.05$), meaning there is no influence on cell viability due to the addition of hBN. The number of cells used for this viability study was 1070 and 3130 for the test and control group, respectively.

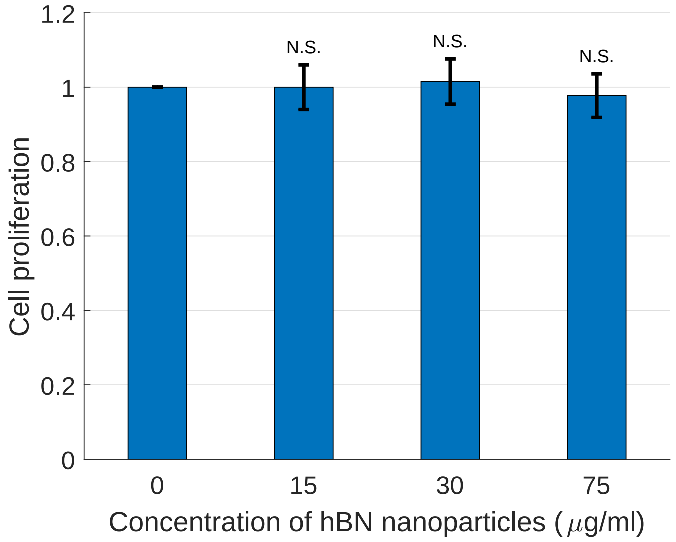

Figure 8: Cellular proliferation over 24 h for different concentrations of hBN nanoparticles normalized to the rate of proliferation for non-treated cells. No statistically significant differences between any of the examples is observed meaning that the cells proliferate with the same rate regardless of the hBN concentration. The number of cells studied ranged from 815–1135 for different hBN concentration. N.S. above bars indicate non-significant difference ($p > 0.05$) between each result and the control based on Two-sample t-test.

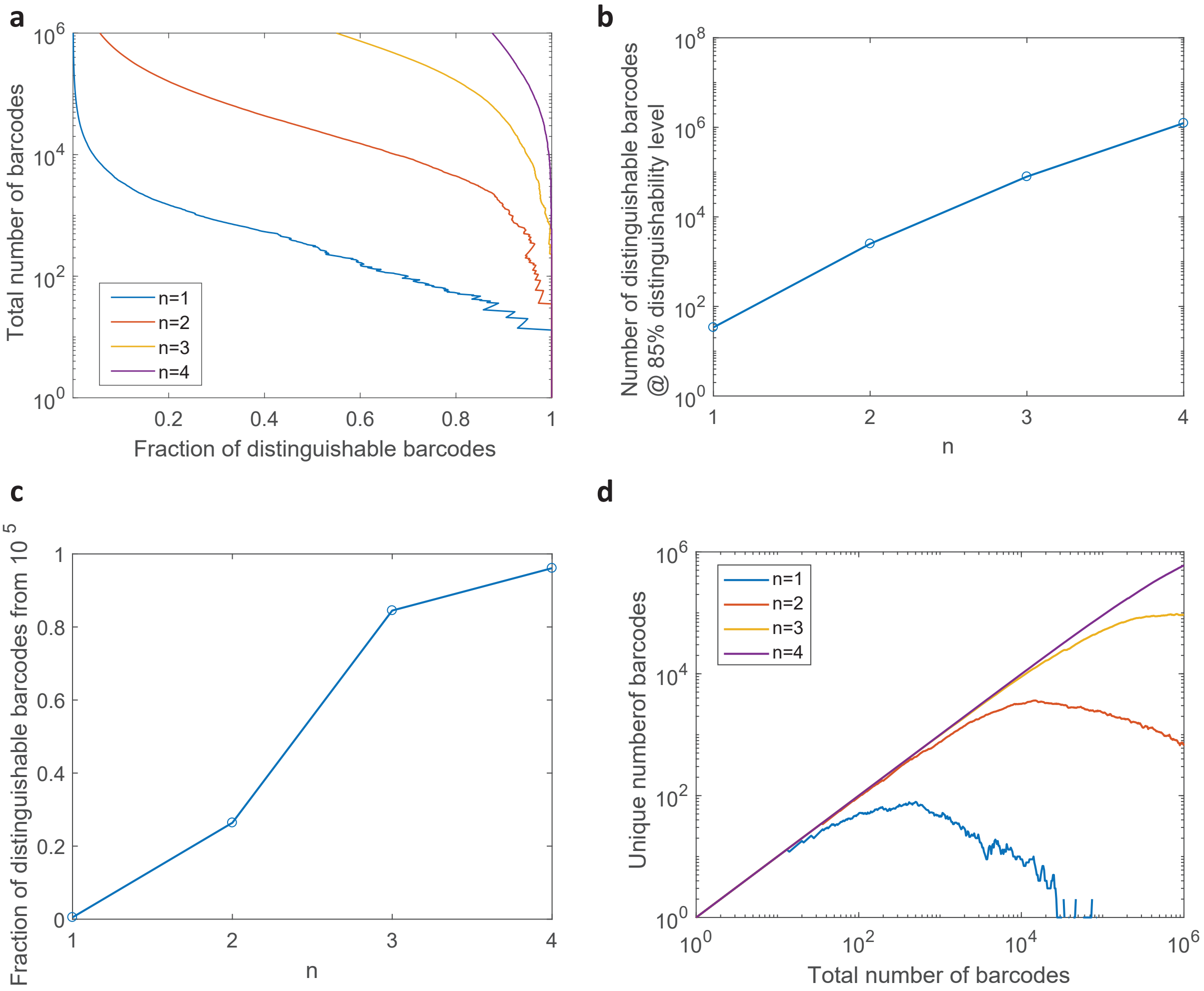

Figure 9: A Monte Carlo calculation of the number of possible spectral barcodes which can be generated by the color centers inside cells. **a)** A calculation of how large is the barcode sample size at a given distinguishability percentage. This would in practice correspond to how many cells can we have in the sample so that a given percentage of them will be distinguished. **b)** Number of distinguishable barcodes at 85% distinguishability level for different n, denoting the number of spectral peaks in each barcode, i.e. number of color centers per cell. The distinguishability level of 85% relates to the percentage of the cells in the sample that can be distinguished. It can be seen that the value is increasing approximately exponentially when raising n. **c)** Alternatively, we can look at a sample of a given size, in this case, 10^5, and determine how the fraction of distinguishable barcodes changes with increasing n. Values of n above 4 would only be needed when one would want to realize well above 10^5 distinguishable barcodes. **d)** Another useful information is how many unique barcodes we can get, meaning that not only are they distinguishable but they also do not have duplicates. For $n = 4$ and above and for practically useful numbers of barcodes up to 10^6 the number of unique and distinguishable barcodes are almost the same.
